# Association of oral health status with the CD4+ cell count in children living with HIV in Phnom Penh, Cambodia

**DOI:** 10.1038/s41598-019-51077-0

**Published:** 2019-10-10

**Authors:** Kimiyo Kikuchi, Yusuke Furukawa, Sovannary Tuot, Khuondyla Pal, Chantheany Huot, Siyan Yi

**Affiliations:** 1 Institute of Decision Science for a Sustainable Society, Kyushu University, Fukuoka, Japan; 2 Section of Orthodontics, Kyushu University Hospital, Fukuoka, Japan; 3 KHANA Center for Population Health Research, Phnom Penh, Cambodia; 4 National Paediatric Hospital, Phnom Penh, Cambodia; 5 Center for Global Health Research, Touro University California, Vallejo, CA USA; 6 Saw Swee Hock School of Public Health, National University of Singapore and National University Health System, Singapore, Singapore

**Keywords:** HIV infections, Dental caries

## Abstract

The association between oral and overall health, and particularly between dental and immune health, in children living with HIV remains unclear. This study examined the association between the decayed, missing and filled teeth (DMFT) score and CD4+ cell counts in 142 children living with HIV aged 8–15 years (male, 51%) from Phnom Penh, Cambodia. Other indicators of oral health (e.g., debris index, salivary flow, salivary pH and oral health-related quality of life) and overall health (e.g., nutritional status and quality of life) were also evaluated. DMFT scores were negatively associated with the CD4+ cell count in male children (β: −0.13, 95% confidence interval [CI]: −0.25, −0.02). In all children, positive associations were observed between salivary pH and CD4+ count (β: −0.645, 95% CI: 0.02, 1.25) and between salivary flow and height-for-age Z-score (β: 1.22, 95% CI: 0.50, 1.95). The debris index was negatively associated with the height-for-age Z-score (β: −2.04, 95% CI: −3.38, −0.71). In summary, oral health was associated with immune and nutritional status. Oral health policies for children living with HIV should be emphasised, and further studies should evaluate the mechanism underlying the relationship between oral and overall health.

## Introduction

In a general population of children, oral health status is an important contributor to the overall health status^1^. Poor oral health is associated with a lower quality of life^2^ and increases in the incidence of some infectious diseases^3^. Hence, the World Health Assembly has recognised the importance of paediatric oral health (resolution WHA60.17)^4^. Dental caries is a common but preventable childhood disease^5^ associated with tooth loss^6^ and an increased risk of chronic diseases (e.g. cardiovascular disease, diabetes) in adulthood^7^. Dental caries are also associated with various manifestations of paediatric malnutrition, including low body weight and height^8,9^ and iron deficiency^10^. These associations may be due to changes in dietary intake (e.g., the quantity and variety of consumed foods) as a consequence of dental pain.

Compared to uninfected children, children living with HIV (CLWH) face increased oral health risks, including the risk of oral lesions^11,12^ and elevated rates of periodontal disease incidence and progression^13,14^. CLWH also have higher decayed, missing and filled teeth (DMFT) scores than HIV-uninfected children^11,12,15,16^. These discrepancies may be consequent to early childhood dental caries, which themselves may have arisen due to inadequate feeding practices intended to address a failure to thrive or the use of some antiretroviral drugs that contain sugar^14,15,17^. Moreover, antiretroviral drugs may delay dental development directly^18^. In other words, the detection and treatment of oral health problems in CLWH are particularly important in terms of maximising the quality and longevity of life^15^.

Currently, the association between the oral and overall health statuses, and particularly between the dental health and immune status, of CLWH remains unclear. Findings from a previous study revealed a significant association between the frequency of HIV-related oral lesions and the immune status^19^. However, few studies have addressed the associations of other oral health indicators, including dental health, salivary status and oral health-related quality of life, with the immune status. Furthermore, the potentially important associations of a child’s oral health status with their nutritional status and quality of life remain largely unexplored. Such associations, if present, might suggest that oral health affects the overall health status.

Oral health is a significant problem affecting children in Cambodia. A previous report demonstrated a higher estimated mean DMFT score among the general Cambodian children relative to the worldwide and Western Pacific Region mean scores^20^. The higher DMFT score in this population may be associated with a sugar intake by female children that exceeds the allowance recommended by the World Health Organisation (WHO)^21,22^. Over time, the number of new HIV infections in Cambodia has decreased, and approximately 4200 CLWH are now thought to reside in the country^23^. Given the severe oral health status of the general children in Cambodia, one might expect CLWH to have a similar or worse oral health status as a consequence of a higher risk of oral health problems in CLWH. Therefore, the main objective of this study was to examine the relationship between the DMFT score and immune status (e.g., CD4+ cell count) among CLWH in Phnom Penh. We also examined the relationship between other oral health indicators (e.g., debris index, salivary flow, salivary pH and oral health-related quality of life score) and overall health indicators (e.g., nutritional status and quality of life) in this population.

## Results

### General characteristics and health status

Initially, data were collected from 151 children. After excluding nine subjects with incomplete or outlier data, 142 subjects were included in the analysis. Table 1 presents the general and health-related characteristics of the included children. Briefly, 51% of the participants were male, and the mean age was 12.3 (standard deviation [SD], 1.8) years. The mean duration on ART was 4.9 (SD, 3.3) years, and the mean CD4+ cell count was 840.1 (SD, 286.1) cells/mm^3^. The children had mean overall health-related quality of life and oral health-related quality of life scores of 75.7 (SD, 12.8) and 15.9 (SD, 8.7), respectively. The most frequently reported HIV-related symptoms were abdominal/stomach pain (62.7%), headache (59.9%) and dry or painful mouth/oral problems (53.8%) The mean DMFT was 4.3 (SD, 3.3). Male children had a significantly lower body mass index (BMI)-for-age, compared to female children (−1.53 vs. −0.93; p < 0.01), as well as a higher debris index score (1.9 vs. 1.6; p < 0.01) and salivary pH (5.8 vs. 5.6; p = 0.03). Moreover, a significantly higher percentage of male children reported experiencing diarrhoea, compared to female children (57.5% vs. 40.6%; p = 0.04).

**Table 1:** General characteristics and health statuses of the participants.

Characteristics	n/mean	%/SD	Female	Male	p-value
**Sex of children**					
Male	73	51.4			
Female	69	48.6			
Age of children (years)	12.3	1.8	12.3	12.3	0.87
<11	21	14.8			
11	22	15.5			
12	30	21.1			
13	26	18.3			
14	27	19.0			
15	16	11.3			
Duration under antiretroviral therapy (years)	4.9	3.3	4.6	5.1	0.30
CD4 + cell count (cells/mm^3^)	840.1	286.9	870.6	811.2	0.22
Body mass index for age	−1.24	1.15	−0.93	−1.53	<0.01**
Normal: 1 to −2SD	110	77.5			
Underweight: <−2SD	23	16.2			
Severely underweight: <−3SD	8	5.6			
Height-for-age	−2.02	1.17	−1.98	−2.06	0.71
Overall health-related quality of life	75.7	12.8	73.8	77.5	0.08
Physical health	77.6	16.8			
Emotional functioning	72.3	17.1			
Social functioning	82.1	16.0			
School functioning	69.6	16.9			
**HIV symptoms**					
Abdominal/stomach pain	89	62.7	65.2	60.3	0.54
Headache	85	59.9	67.1	53.4	0.094
Dry or painful mouth/trouble swallowing	77	53.8	59.4	49.3	0.23
Physical or bodily pain	72	50.7	48.5	53.4	0.56
Nausea/vomiting	73	51.0	47.8	54.8	0.41
Diarrhoea	70	49.0	40.6	57.5	0.04*
Dizziness/light-headedness	58	40.6	44.9	37.0	0.34
Fevers/night sweats/shaking/chills	54	38.0	44.9	31.5	0.10
Chest pain/tightness	46	32.4	29.0	35.6	0.40
DMFT score	4.3	3.3	4.5	4.2	0.54
Debris index	1.8	0.5	1.6	1.9	<0.01**
Salivary pH	5.7	0.6	5.6	5.8	0.03*
Salivary flow (mL/min)	0.5	0.3	0.5	0.5	0.59
Oral health-related quality of life	15.9	8.7	16.6	15.1	0.32

SD: standard deviation; DMFT: decayed, missing and filled teeth.

*p < 0.05; **p < 0.01.

### Associations between indicators of oral health and overall health

Overall, a positive association was detected between a higher salivary pH and higher CD4 count (β: 0.65, 95% confidence interval [CI]: 0.02, 1.25). In a sex-stratified analysis, the DMFT score was negatively associated with the CD4+ cell count only in male children (β: −0.13, 95% CI: −0.25, −0.02).

Table 2 presents the associations between different oral health indicators and height-for-age Z-scores (HAZ) among all children. Notably, the debris index score was shown to associate negatively with the HAZ score (β: −2.04, 95% CI: −3.38, −0.71), while the salivary flow was shown to associate positively with this parameter (β: 1.22, 95% CI: 0.50, 1.95). Table 3 presents the associations between different oral health indicators and the overall health-related quality of life. A higher oral health-related quality of life score was shown to associate negatively with a higher overall health-related quality of life (β: −0.17, 95% CI: −0.19, −0.10).

**Table 2:** Associations of oral health indicators with height-for-age among all participants.

	Coefficient	95% CI		Coefficient	95% CI		Coefficient	95% CI		Coefficient	95% CI		Coefficient	95% CI	
DMFT score	−0.20	−0.91	0.52												
Debris index				−2.04	−3.38	−0.71**									
Salivary pH							4.05	−0.01	8.10						
Salivary flow										1.22	0.50	1.95**			
Oral health-related quality of life													−0.50	−1.17	0.19
Age	−0.06	−0.19	0.07	−0.09	−0.21	0.03	−0.04	−0.15	0.08	−0.04	−0.16	0.07	−0.07	−0.19	0.05
Sex	−0.15	−0.58	0.28	0.09	−0.31	0.49	−0.14	−0.53	0.26	−0.05	−0.43	0.33	−0.10	−0.50	0.29
Duration under ART	−0.01	−0.59	0.56	−0.05	−0.58	0.48	−0.02	−0.56	0.52	−0.10	−0.62	0.43	−0.04	−0.59	0.50

CI: confidence interval; DMFT: decayed, missing and filled teeth; ART: antiretroviral therapy.

*p < 0.05; **p < 0.01.

**Table 3:** Associations of oral health indicators with the overall health-related quality of life among all participants.

	Coefficient	95% CI		Coefficient	95% CI		Coefficient	95% CI		Coefficient	95% CI		Coefficient	95% CI	
DMFT score	<0.01	−0.05	0.06												
Debris index				0.03	−0.08	0.13									
Salivary pH							−0.02	−0.33	0.28						
Salivary flow										−0.02	−0.08	0.04			
Oral health−related quality of life													−0.17	−0.19	−0.10*
Age	<0.01	− <0.01	0.01	<0.01	−0.01	0.01	<0.01	−0.01	0.01	<0.01	−0.01	0.01	− <0.01	−0.01	<0.01
Sex	0.03	− <0.01	0.06	0.02	−0.01	0.05	0.03	−0.01	0.05	0.02	−0.01	0.05	0.01	−0.01	0.04
Duration under ART	<0.01	−0.05	0.06	−0.01	−0.05	0.04	−0.01	−0.05	0.03	−0.01	−0.05	0.04	−0.01	−0.05	0.02

CI: confidence interval; DMFT: decayed, missing and filled teeth; ART: antiretroviral therapy.

*p < 0.01.

## Discussion

Our study of CLWH in Phnom Penh, Cambodia, yielded several interesting findings. We detected a positive association of the salivary pH with the CD4+ cell count in both male and female participants. However, we only observed a negative association of the DMFT score with the CD4+ cell count in male participants. These observations suggest that a higher salivary pH level, an essential factor in the prevention of dental caries, is associated with a better immune status, while a worse dental health status is closely associated with a decreased immune status. The former observation is consistent with the findings of a previous study^24^. Notably, reduced immunity or immunodeficiency may lead to a decrease in salivary flow, which may impede the recovery of the salivary pH level after eating^25^. Similar findings have been observed in patients with other diseases of immunodeficiency, such as end-stage renal disease^25^.

The latter observation, namely the association of the DMFT score with CD4+ cell count in male participants, was also consistent with the findings of a previous study of CLWH aged 2–9 years which revealed a negative association between these factors. However, that study included children categorised as severely immune-suppressed (CD4+ count <200 cells/mm^3^)^26^. A very similar result was reported among children with advanced-stage disease who participated in another study of CLWH aged 0.3–14 years^16^. In yet another study, CLWH aged 2–14 years who exhibited advanced immune suppression (200–349 CD4+ cells/mm^3^) had higher DMFT scores, although this association was not significant^27^. Similar to our observations, those study findings suggest that a lower level of immunity is closely associated with a worse dental health status. This may be attributable to the presence of xerostomia, which is often diagnosed due to a lack of salivary flow in people living with HIV who present with advanced immune suppression^25,28^. As mentioned above, reduced salivary flow hinders the recovery of the salivary pH level and thus renders the oral cavity favourable to dental caries.

As noted, the association between the DMFT score and CD4+ cell count was identified only among male participants in our study, possibly as a consequence of sex-related physiological differences^29^. In terms of dental development, permanent teeth generally erupt earlier in female children, and thus male children face a later onset of the risk of caries. Furthermore, female children usually undergo menarche at approximately 10–13 years of age, and the resulting hormonal imbalances reduce their level of immunity^30^. In our study, male participants had a significantly lower BMI-for-age than female participants (p < 0.01), suggesting a delay in the physical development of male children that might have also led to a delay in dental development specifically among male children. Additionally, male participants had significantly higher debris index scores, compared to female participants (p < 0.01), which would also suggest a sex-related difference in the caries risk that may have contributed to the difference in results between male and female children.

The results of this study also revealed the associations of the salivary flow and debris index scores with the HAZ, suggesting that a better oral health status is associated with a better nutritional status. A similar observation was reported from a study of the general populations, in which malnutrition during childhood was associated with a lower salivary pH and reduced salivary flow during adolescence^31^. According to that study, chronic postnatal malnutrition throughout childhood might have a mechanistic effect at the molecular level that would prevent age-appropriate physical and salivary gland development, resulting in the ineffective removal of dental plaque^32^. Such a molecular mechanism might also explain our observation that a high debris index score was associated with a lower HAZ. In general, perinatally HIV-infected children have worse nutritional status than uninfected children, and our study population was no exception. Specifically, our participants had a mean HAZ score that was categorised as ‘stunting’ (HAZ-score <−2.00). Therefore, CLWH could be considered at risk of delayed salivary gland development and, consequently, at risk of a worse oral health status. Oral care should be promoted and provided to these children to compensate for the disadvantages of an impaired oral health status.

Our findings suggest that oral and overall health are closely related in CLWH. Possibly, an underlying mechanism exists by which HIV-induced advanced immune suppression yields an oral environment favourable for the reproduction of cariogenic bacteria^26^. However, the findings of other studies have suggested the opposite, namely that oral health influences overall health^3,33^. This is particularly true with respect to periodontal disease-causing bacteria, which are known to trigger the onset of acquired immune deficiency syndrome (AIDS)^34,35^. Further research is needed to clarify the influence of oral health status on immune status in HIV-infected populations. Moreover, intervention studies are needed to demonstrate whether an improved oral health status can prevent the onset of AIDS or improve the overall health status.

This study has some limitations. The cross-sectional design did not enable us to prove causality. We also did not examine changes in oral microbial colonisation and the effect of HIV on cariogenic bacteria in the participating children. Therefore, additional studies are needed to determine which factors affect the risk of dental caries in this population. Furthermore, the use of a single-centre setting may have caused an inherent sampling bias. However, Phnom Penh is home to the largest number of CLWH in Cambodia, and the research site is the only referral hospital for CLWH within Phnom Penh. Therefore, our findings may be significant and applicable to the general population of CLWH in Cambodia.

In conclusion, this study demonstrated associations of a better oral health status with better immune and nutritional status in CLWH in Cambodia. Our findings underscore the importance of promoting oral health policies for CLWH. Further research is needed to clarify the mechanism between the oral and overall health status in this population.

## Methods

### Study design and site

We conducted a cross-sectional study at the National Paediatric Hospital in Phnom Penh in 2017. This study setting was selected because most CLWH in Phnom Penh and the surrounding regions undergo treatment at this hospital. Furthermore, this hospital includes all diagnostic and therapeutic paediatric departments, including antiretroviral therapy (ART) and dental clinic. The majority of children treated at this hospital were provided antiretroviral drugs such as zidovudine, lamivudine and nevirapine, which were available in the form of syrups or tablets. The sucrose contained in these syrups is often considered to have cariogenic potential^17^. The overall framework of the study included interviews of both CLWH and their caregivers. We analysed the children’s data related to their quality of life and health examinations, as well as medical record data.

### Participants

CLWH aged 8–15 years were included in the analyses. The inclusion criteria were the receipt of care and treatment at the study site hospital and a duration under ART of at least 3 months. We excluded children who were unable to respond to the interview items because of mental or physical illness. Regarding the selection, we initially selected children aged 8–15 years from the list of registered CLWH at the hospital. Next, a random number table was used to select candidates for study participation. The selected children were interviewed until the necessary sample size was achieved. Randomisation was performed by research assistants other than the main researchers. We did not include HIV-uninfected children as a control population because the aetiologies underlying a change in the CD4+ cell count may vary between CLWH and uninfected children. Particularly, in CLWH, these counts may change due to HIV or ART and therefore cannot be compared with respective changes in uninfected children.

### Sample size calculation

Following a previous Cambodian study^36^, which reported the prevalence of dental caries as 48% among 12-year-old children, a minimum total sample of 150 participants was required. Statistical significance and power were set at 0.05 and 0.80, respectively. The value included a 10% surplus to accommodate invalid responses.

### Data collection

We conducted face-to-face interviews of CLWH using a structured questionnaire. We also collected data on the children’s oral health status, body weight and height after the interviews, as well as medical record data such as the most recent CD4+ cell count, date of birth, history of opportunistic infectious diseases and date of ART regimen initiation.

Prior to the interview process, six research assistants participated in a 1-day training session intended to increase their understanding of the questionnaires. Subsequently, the research assistants administered the interviews to 10 participants not included in the main study as a pre-test.

### Questionnaire

We developed the interview questionnaire based on previous studies^37–39^. The questionnaire comprised questions concerning the children’s socio-demographic characteristics, overall health-related quality of life and oral health-related quality of life.

The overall health-related quality of life was assessed using the Paediatric Quality of Life inventory (PedsQL^TM^ 4.0)^40^, which was previously validated for CLWH^41^. The PedsQL^TM^ 4.0 comprises 23 items, and the response options for each item are measured on a five-point scale ranging from ‘almost always’ (0) to ‘never’ (4). The items were transformed linearly to a 0–100 scale as follows: 0 = 100, 1 = 75, 2 = 50, 3 = 25, and 4 = 0. A higher score indicates a higher overall health-related quality of life. The Cronbach’s alpha was 0.77 among participants in this study.

The oral health-related quality of life was assessed using the Child Perceptions Scale (i.e., Child Perceptions Questionnaire)^42^, which has been validated in a Cambodian setting^42,43^. This scale comprises 16 questions for which the response options were measured on a five-point scale ranging from ‘never’ (0) to ‘every day or almost every day’ (4). A higher score indicates a lower oral health-related quality of life. The Cronbach’s alpha was 0.81 among the participants in this study.

### Oral health

We collected information about the children’s dental status, dental plaque, salivary pH, and salivary flow through dental examinations and saliva tests. Regarding the dental status, we determined the DMFT score of each child by observing the teeth directly and calculating the number of teeth that were decayed, missing or filled. The dentition status of each child was recorded on a WHO oral health assessment form^37^. A higher DMFT score indicates a worse dental status. We also examined the presence of dental plaque by staining the children’s teeth with a plaque-disclosing gel (Dentclub; Niimikagaku kogyo, Gunma, Japan). The degree of staining of each tooth was scored using a range from 0 to 3 (0 = no stain, 3 = fully stained). A debris index score was then calculated based on the extent of staining on the dental surfaces, with a higher score indicating a worse plaque status. All dental status and dental plaque data were collected by a dentist who participated in the research team. To increase reproducibility and assure calibration, one dentist examined the dentition status of all children according to the WHO guidelines^37^. Furthermore, the stained teeth were photographed to enable later verification of the scoring accuracy. The salivary pH was assessed using a saliva test kit (CAT21 Buf; Morita, Osaka, Japan) and was recorded in values of pH 4.0 to pH 6.5. The salivary flow was assessed as the total production of saliva in 3 minutes without stimulation (i.e., chewing gum).

### Body weight and height

We measured the body weight (kg) and height (cm) of each child using electronic scales (Omron, Kyoto, Japan) and a manual stadiometer, which were calibrated to 0.1 kg and 0.1 cm, respectively. The HAZ and BMI-for-age were calculated using AnthroPlus software (WHO; http://www.who.int/growthref/tools/en/).

### Statistical analyses

First, the data were stratified by sex. Differences in the general characteristics and health statuses of male and female participants were determined using the chi-square test for categorical variables and Student’s t-test for continuous variables. Next, we conducted a multiple linear regression analysis of the associations between oral health indicators and overall health indicators in the participating children. The oral health indicators included the DMFT score, debris index, salivary flow, salivary pH and oral health-related quality of life score. The overall health indicators included the CD4+ cell count, HAZ, BMI-for-age and overall health-related quality of life score. The age and sex of the child and duration of ART therapy were included as covariates. Statistical significance was set at a two-tailed P value < 0.05. Variables that exhibited multi-collinearity were excluded from the analyses. Statistical software (SPSS version 24.0; IBM Corp., Armonk, NY, USA) was used for data entry and analyses.

### Ethics approval and consent to participate

We obtained ethical approval from the Research Ethics Committee of Kyushu University (28–378) and the National Ethical Committee for Health Research, Ministry of Health, Cambodia (022NECHR). All experimental procedures were performed in accordance with the relevant guidelines and regulations. We obtained informed consent from the children’s parents and/or legal caregivers prior to data collection and subsequently obtained assent to participate in the study from the children. All participation was voluntary, and the confidentiality of the subjects was maintained.

## Data Availability

The datasets used and/or analysed in the current study are available from the corresponding author upon reasonable request.
